# Metabolic Degradation of 1,4-dichloronaphthalene by *Pseudomonas* sp. HY

**DOI:** 10.3390/ijerph120910300

**Published:** 2015-08-25

**Authors:** Jian Yu, Xiaoli Wu, Youqun Song, Wenhui Ren, Hao L. Tang

**Affiliations:** 1 Department of Water Engineering and Science, College of Civil Engineering, Hunan University, Changsha 410082, China; E-Mail: yujianpro@126.com; 2 Guangzhou Water Supply Co., Guangzhou 510600, China; E-Mail: willy-427@163.com; 3 State Key Laboratory of Chemo/Biosensing and Chemometrics, Hunan University, Changsha 410082, China; E-Mail: songyouqun@163.com; 4 Minn Water LLC, Minneapolis, MN 55441, USA

**Keywords:** 1,4-dichloronaphthalene, metabolism, metabolite

## Abstract

There is increasing concern regarding the adverse health effects of polychlorinated naphthalenes (PCNs). The metabolic degradation of 1,4-dichloronaphthalene (1,4-DCN) as a model PCN, was studied using a strain of *Pseudomonas* sp. HY. The metabolites were analyzed by gas chromatography-mass spectrometry (GC-MS). A series of metabolites including dihydroxy-dichloro-naphthalene, epoxy-dichlorinated naphthalene, dichlorinated naphthol, and dichlorinated salicylic acid were identified. The time-concentration plots of the degradation curves of 1,4-DCN was also obtained from the experiments, which set the initial concentration of 1,4-DCN to 10 mg/L and 20 mg/L, respectively. The results showed that 98% removal could be achieved within 48 h at an initial 1,4-DCN concentration of 10 mg/L. Nevertheless, it took 144 h to reach the same degradation efficiency at an initial concentration of 20 mg/L. The degradation of 1,4-DCN may not remove the chloride ions during the processes and the metabolites may not benefit the bacterial growth. The research suggests a metabolic pathway of 1,4-DCN, which is critical for the treatment of this compound through biological processes.

## 1. Introduction

1,4-dichloronaphthalene (1,4-DCN) is one of polychlorinated naphthalenes (PCNs)–a class of chlorinated aromatic hydrocarbons, which were highly hydrophobic, semi-volatile, thermally stable, and electrical insulating. PCNs can be used in engine oil additives, heat exchange fluids, flame retardants, while most uses were found in cable insulation and wood preservatives [1]. PCNs have been detected in water and sediments receiving industrial and municipal sewer discharges, and concentrations of up to 73 µg/L in polluted water [2] and 61 mg/kg dry weight in sediments [3] were detected, respectively. PCNs were toxic [4], resistant to degradation, and they can travel long distances and build up in the bodies of plants and animals, causing a danger to human health and environment. They were therefore included in the list of persistent organic pollutants (POPs) candidates. PCNs from trichloronaphthalene to octachloronaphthalene were also included in the list of priority pollutants by Convention for the Protection of the Marine Environment of the North-East Atlantic (OSPAR Convention) [5]. Because of the detection in human adipose tissue, liver, blood, and breast milk at concentrations in the ng/kg lipid range, there is a growing concern regarding their adverse health effects [6]. Severe skin reactions and liver disease have both been reported after occupational exposure to PCNs and the laboratory mammals’ tests have correlated them with genotoxicity and reproductive toxicity [3]. It is therefore crucial to investigate an effective approach for PCN removal.

To date, most studies on the microbial degradation of PCNs were focused on the degradation of monochloronaphthalenes. There is little information on the microbial degradation of DCNs. It was reported that a strain of naphthalene-degrading bacteria isolated from soil could cometabolize 1-chloronaphthalene and 2-chloronaphthalene. The metabolic intermediates of 1-chloronaphthalene was 1,2-dihydroxy-1,2-dihydro-8-chloronaphthalene and 3-chloro-salicylic acid, and that of 2-chloronaphthalene was 1-chloro-2-hydroxy-oxohexadienoate [7]. The degradation of 1-chloronaphthalene and 2-chloronaphthalene by white-rot fungi showed that 1-chloronaphthalene was removed by 80% in 24 h at 30 °C, when the initial concentration of 1-chloronaphthalene and 2-chloronaphthalene was 100 µM. While 1-chloronaphthalene and 2-chloronaphthalene were added simultaneously, the removals achieved 74% and 94%, respectively, in 24 h [8]. Durham and Steward [9] found a strain of *Pseudomonas putida* could oxidize 1,4-DCN, and the rate of the intermediate 3,6-dichlorosalicylate formation was less than 1% of the rate of salicylate formation from unsubstituted naphthalene. Mori *et al.* [10] found that 1,4-DCN could be metabolized by the white-rot fungus *Phlebia lindtneri* to form six metabolites: four putative hydroxylated and two dihydro-dihydroxylated compounds, and one of the hydroxylated products was identified as 2,4-dichloro-1-naphthol. Further investigations on metabolic degradation of DCNs are needed.

To explore the degradation of 1,4-DCN, a dominant bacterial strain (named HY) [11], which can utilize naphthalene as carbon and energy source, was isolated from the activated sludge of a landfill leachate treatment plant and enriched for this study. The objectives of this research were (1) to explore the biodegradability and the microbial metabolites of 1,4-DCN; and (2) to propose the metabolic pathway of 1,4-DCN. The research is critical for the treatment of persistent organic pollutants through microbial processes.

## 2. Experimental Section 

### 2.1. Chemicals and Reagents

The HPLC grade 1,4-DCN and naphthalene were purchased from J&K Scientific Ltd. (Beijing, China), International Laboratory USA, and Sinopharm Group Co. Ltd. (Shanghai, China), respectively. The silylation and derivatization reagent was a mixture of 99% N,O-bi (trimethylsilyl) trifluoro—acetamide (BSTFA) and 1% dimethylchlorosilane (DMCS), purchased from Supleco, Inc (Bellefonte, PA). All other organic solvents and inorganic chemicals were analytical grade, purchased from Sinopharm Group Co. Ltd. (Shanghai, China). 

### 2.2. Preparation of Bacterial Strain Pseudomonas sp. HY

Three solutions were prepared as follows: Solution A: 6.77 g/L K_2_HPO_4_, 21.94 g/L KH_2_PO_4_, 12.96 g/L (NH_4_)_2_SO_4_; Solution B: 9 g/L MgSO_4_, 5 g/L MnSO_4_·H_2_O, 1 g/L FeSO_4_·7H_2_O, 0.5 g/L CaCO_3_·2H_2_O; Solution C: 1.44 g/L ZnSO_4_·7H_2_O, 0.25 g/L CuSO_4_·5H_2_O, 0.28 g/L CoSO_4_·7H_2_O. The inorganic culture media was prepared by mixing 77.5 mL solution A, 1 mL solution C, and 910 mL distilled water, followed by sterilization at 120 °C for 20 min, addition of 10 mL sterilized Solution B, and final adjustment of pH to 7.

The bacterial strain *Pseudomonas* sp. HY was isolated from the activated sludge of a landfill leachate treatment plant following protocols described by Yu *et al.* [11]. In short, 1 mL of the sludge supernatant was transferred to an inorganic culture media containing 1000 mg/L naphthalene and 10 mg/L 1,4-DCN. During a two-month incubation period (pH 7, 180 rpm, 25 °C), the concentration of 1,4-DCN was gradually increased from 10 to 80 mg/L and the concentration of naphthalene was decreased from 1000 to 50 mg/L while 10% of the enrichment culture was transferred to fresh media every 8–10 days. The pour plate method was used to exam the morphology of the bacteria.

The bacteria were then inoculated to the inorganic culture media containing 1000 mg/L nathphalene and incubated at 25 °C in absence of light until the bacterial population approached the end of its exponential growth phase. The naphthalene particles in the culture media were then removed by glass wool filtration. A 0.05 M KH_2_PO_4_-KOH buffer solution was used to recover the bacteria for the experiments.

### 2.3. Degradation Experiments

To meet the oxygen demand for microbial growth, 40 mL glass vials were used for degradation experiments [12]. The procedures were as follows: the N-hexane with 1,4-DCN solution was added into the 40 mL vials. It took a few seconds for N-hexane to evaporate; then 5 mL sterilized inorganic culture media was added, followed by addition of the bacterial solution. The optical density at 600 nm (OD_600_) was then adjusted to 0.25. Vials were sealed with Teflon caps and incubated in absence of light under 25 °C, 180 rpm, and pH 7. Samples of the culture solutions were taken periodically and analyzed for 1,4-DCN residuals and OD_600_ values. Blank samples as negative no-bacteria control were 1:1 HCl solutions with pH < 2. All degradation experiments were conducted in parallel and the results reported were arithmetical average values of duplicate sets.

### 2.4. 1,4-DCN Analysis

A volume of 5 mL sample was processed for Gas Chromatography (GC) analysis. 5 mL N-hexane and 2 g anhydrous sodium sulfate were added to each sample. The samples were sealed with Teflon caps and agitated at 2000 rpm for 10 min. After 5-min stratification, the organic layer of each sample was transferred to a GC vial. The extraction process was completed at 25 °C or above to avoid sample emulsification. The GC (Model GC-14C, Shimazu Co. Ltd., Kyoto, Japan) used a DB-5 capillary column. The temperature ramping program was as follows: Initial at 160 °C for 2 min, ramp to 270 °C at 10 °C per minute, and hold for 3 min. The sample intake amount was 1 µL and the split ratio was 1:10. The recoveries of 1,4-DCN ranged from 90% to 110%. The degradation efficiencies of 1,4-DCN were calculated as follows:

(1) $$Degradation\;Efficiency=\;\frac{C_{0}-C_{t}}{C_{0}}\times 100\%$$

where *C_0_* was the initial 1,4-DCN concentration and *C_t_* was the residual 1,4-DCN at time *t*, respectively.

### 2.5. Metabolite Analysis

A volume of 135 mL sample of the metabolites was taken at 8 h, 24 h, and 72 h, respectively. Samples were centrifuged at 18,000 rpm for 10 min. After stratification, the supernatant of each sample was collected, adjusted to pH 2–3 by HCl, and enriched using a merck EN column. The column was then eluated with 3 mL eluant (methanol: ethyl acetate: n-hexane = 1:1:1). The eluate was dried by nitrogen gas, quantified to 0.1 mL by ethyl acetate, dehydrated by anhydrous sodium sulfate, and then analyzed by gas chromatography-mass spectrometry (GC-MS). In addition, another set of samples was prepared and added with 0.2 mL silane derivative reagents. The reactions took 20 h at room temperature and the products were analyzed by GC-MS. Filtered samples were analyzed for chloride by ion chromatography (IC) [13].

A Thermo Finnigan Polaris GC/MS system was used for the detection of metabolites with the mass spectral library. The temperature ramping program was as follows: Initial at 100 °C for 3 min, ramp to 270 °C at 5 °C per minute, and hold for 10 min. The sample intake amount was 1 µL, and the ion source temperature was 200 °C. The scanning time was 0.6 second and the scanning range was 40–400 amu. The interface temperature was 250 °C and the emission current was 200 µA.

## 3. Results and Discussion

### 3.1. Metabolic Degradation of 1,4-DCN

**Figure 1:**
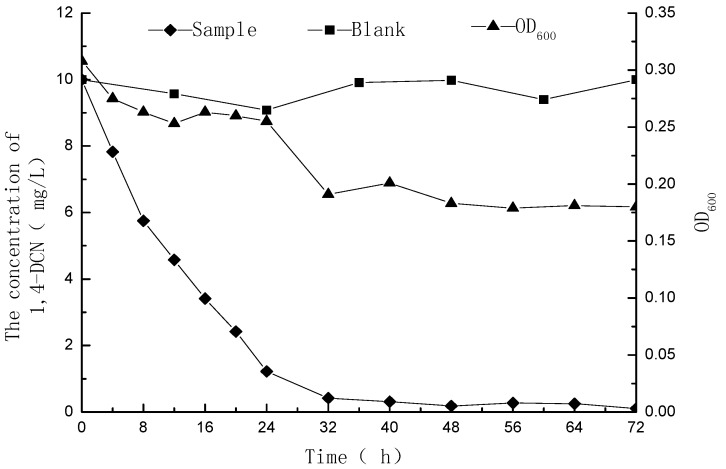
Microbial degradation of 1,4-DCN at an initial concentration of 10 mg/L.

**Figure 2:**
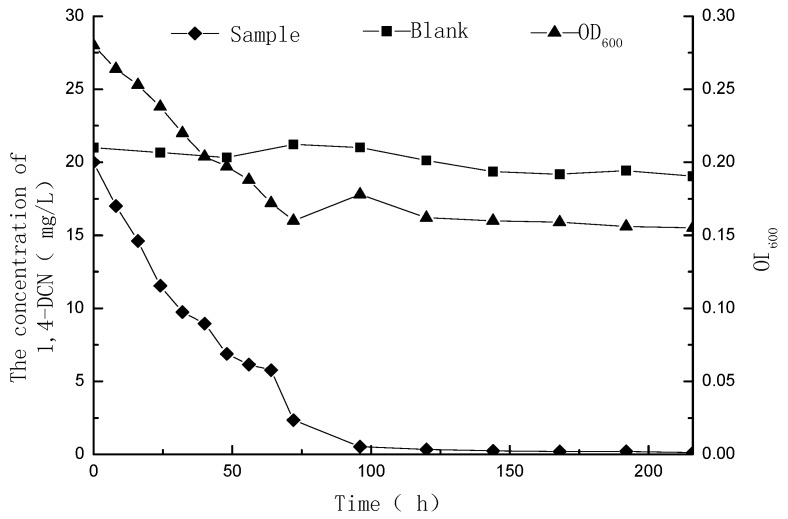
Microbial degradation of 1,4-DCN at an initial concentration of 20 mg/L.

The 1,4-DCN solutions with initial concentrations of 10 and 20 mg/L were examined for residual concentrations and OD_600_ values at different time, and the time-concentration curves were plotted as shown in Figure 1 and Figure 2. The results showed that 98% degradation could be achieved within 48 h while the initial concentration of 1,4-DCN was 10 mg/L. However, it took 144 h to reach the same degradation efficiency at an initial concentration of 20 mg/L. The reductions of OD_600_ at initial 1,4-DCN concentrations of 10 and 20 mg/L were 44% and 38%, respectively. Because the OD_600_ values of the culture media kept decreasing and these values were indicators of bacterial growth [14,15,16], the results imply that the degradation of 1,4-DCN may not benefit the bacterial growth.

### 3.2. Metabolites of 1,4-DCN

**Figure 3:**
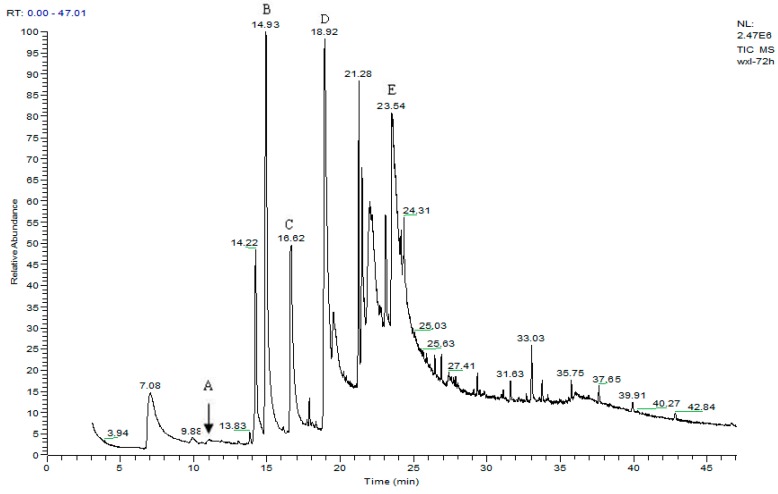
GC spectrum of a 72 h metabolite sample (A. dichlorinated solicylic acid; B. 1,4-dichloronaphthalene; C. dichlorinated salicylic acid; D. epoxy chlorinated naphthalene; E. dichlorinated naphthol).

**Figure 4:**
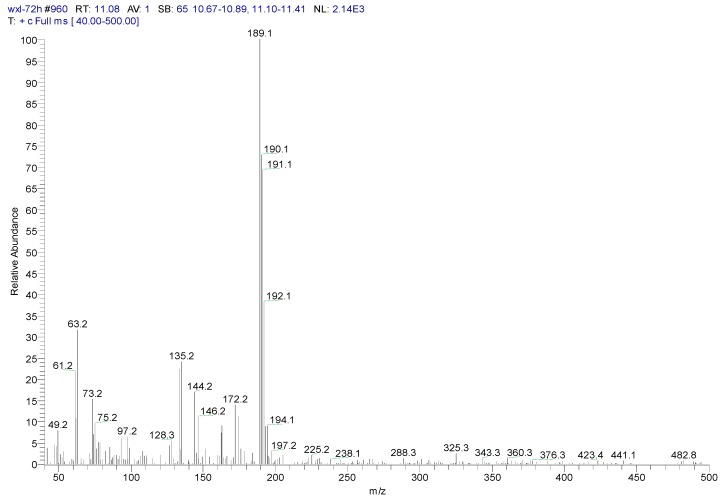
MS spectrum of dichlorinated salicylic aldehyde.

**Figure 5:**
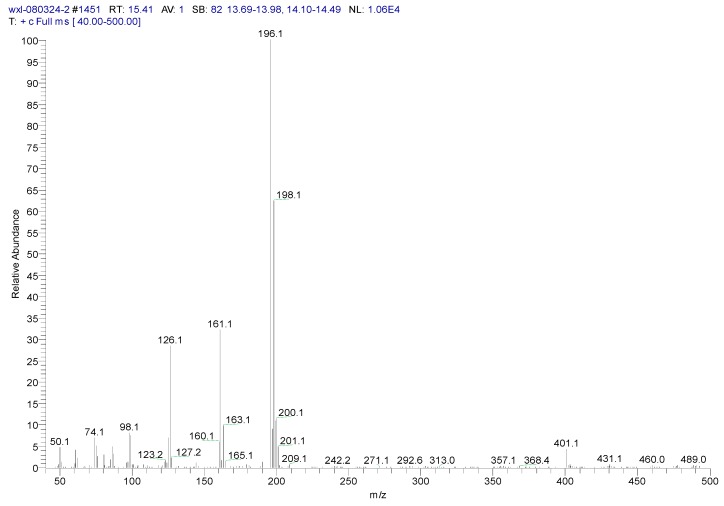
MS spectrum of 1,4-DCN.

**Figure 6:**
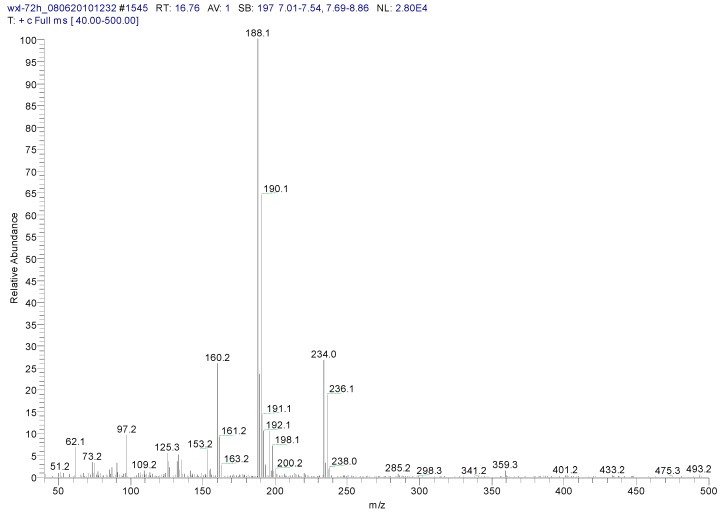
MS spectrum of chlorinated salicylic acid.

**Figure 7:**
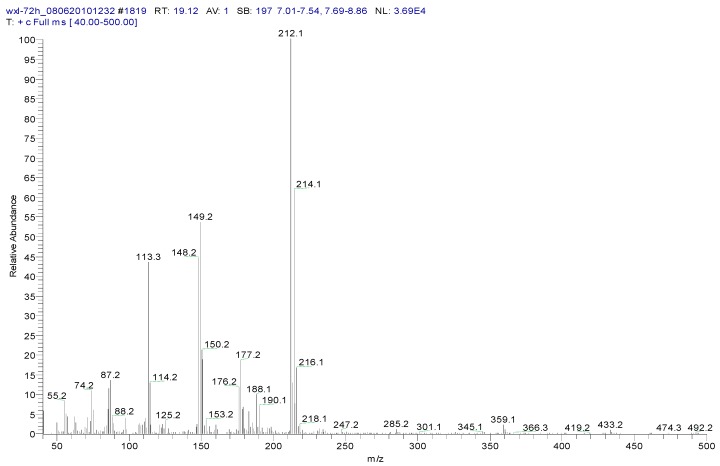
MS spectrum of epoxy-chlorinated naphthalene.

**Figure 8:**
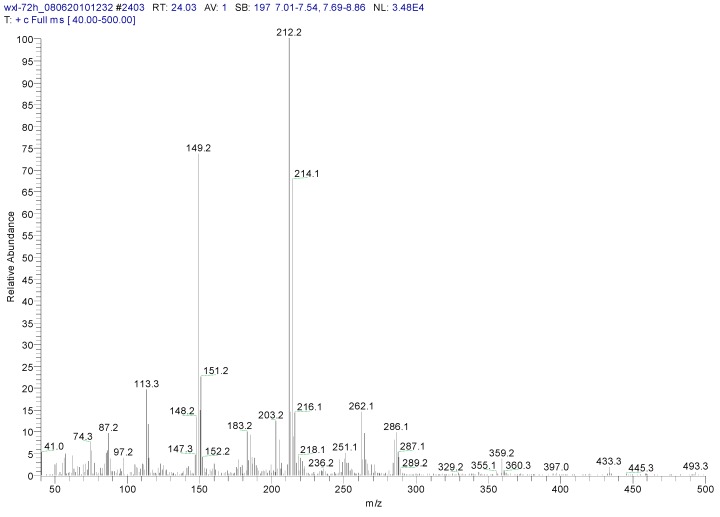
MS spectrum of dichlorinated naphthol.

Metabolite samples of 1,4-DCN at an initial concentration of 10 mg/L were collected at 8 h, 24 h, and 72 h, respectively, during the incubation. After sample centrifugation, C18 enrichment, methanol elution and concentration (Volume reduction was 450 times after drying by nitrogen gas), the samples were analyzed by IC and GC-MS. The IC results revealed no chloride ions in all metabolite samples, indicating that chloride was not removed during the degradation of 1,4-DCN. The GC-MS spectrum of the 72 h sample was shown in Figure 3. According to microbial metabolic pathways of 1-chloronaphthalene, 2-chloronaphthalene, and naphthalene, it was suspected that the metabolites of 1,4-DCN could contain epoxy-dichlorinated naphthalene, dihydroxy-dichlorinated naphthalene, and dichlorinated salicylic acid. The library of GC-MS spectra revealed that the peak at 11.08 min was dichlorinated salicylic aldehyde (m/z 189, 172, 144, 97, 73, 63), the peak at 14.93 min was 1,4-DCN (m/z 196, 161, 126), the peak at 16.628 min was dichlorinated salicylic acid (m/z 188, 160, 153, 125, 97, 73, 62), the peak at 19.92 min was epoxy-chlorinated naphthalene (m/z 212, 149, 113), and the peak at 23.54 min was dichlorinated naphthol (m/z 212, 183, 149, 113, 97, 87, 74), as shown in Figure 3. The GC-MS spectra of these compounds were shown in Figure 4, Figure 5, Figure 6, Figure 7 and Figure 8. While the spectra of metabolite samples collected at different times were compared, almost all of these metabolites were detected, and their concentrations decreased with time, indicating that the metabolites could be degraded by the bacteria. The only exception was dichlorinated salicylic aldehyde, which was only detected in the 72 h samples.

**Figure 9:**
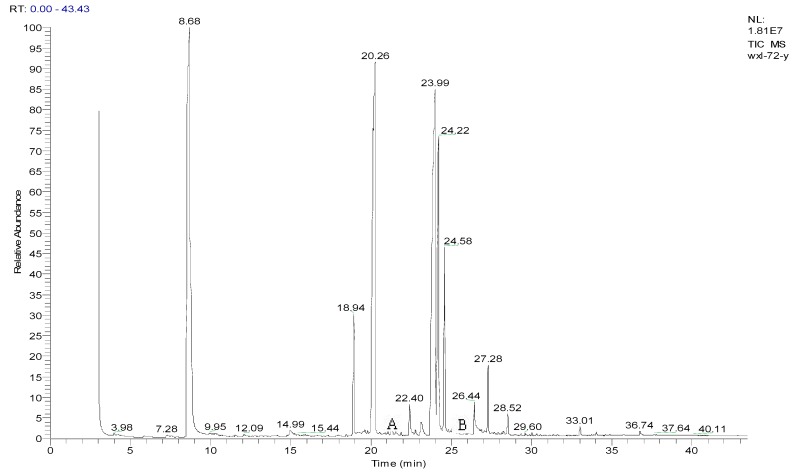
GC spectrum of a silane-derivatized sample (A. the silane derivatives of dichlorinated naphthol; B. the silane derivatives of dihydroxyl dichlorinated naphthalene).

**Figure 10:**
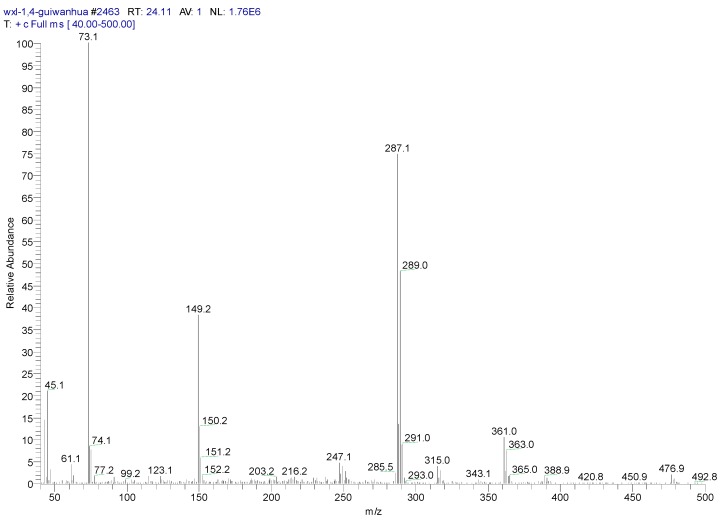
MS spectrum of a silane-derivatized sample at peak 24.41 min.

**Figure 11:**
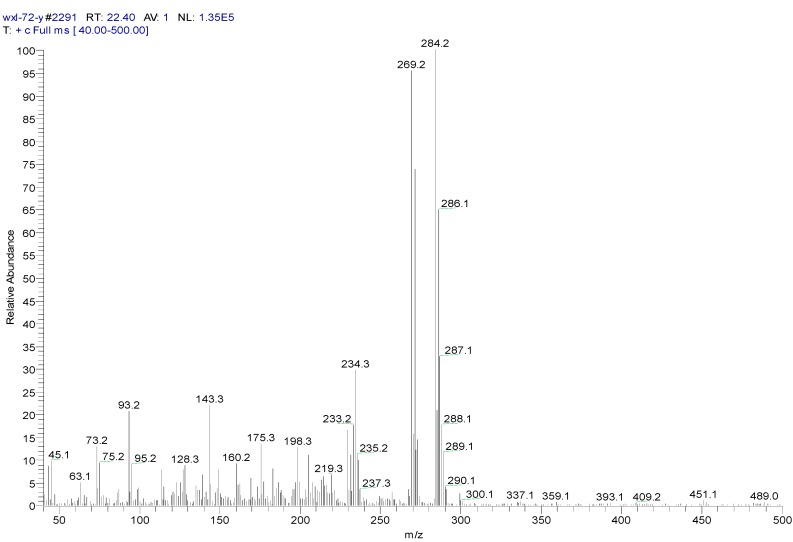
MS spectrum of a silane-derivatized sample at peak 22.4 min.

**Figure 12:**
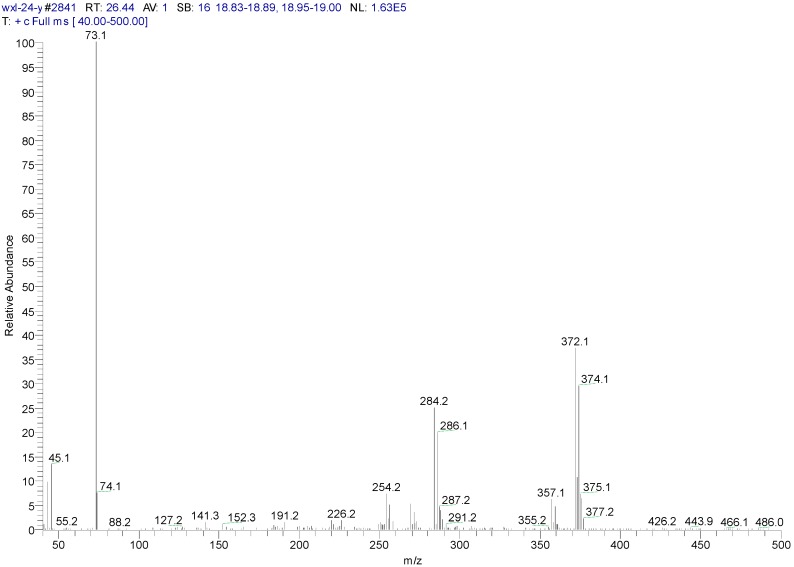
MS spectrum of a silane-derivatized sample at peak 26.4 min.

The metabolites of 1,4-DCN may contain hydroxyl and carboxyl compounds, which were less sensitive to the analysis, although the samples had been highly concentrated. To further identify these compounds, silane derivatization was conducted on the samples. The GC spectrum of a silane-derivatized sample was shown in Figure 9, and the MS spectrum of the peak at 24.11 min was shown in Figure 10. The largest piece was m/z 287, which was likely a metabolite of dihydroxy-dichlorinated naphthol (molecular weight (MW) = 302) after removing a –CH3 (MW = 15). The isotope abundance distribution also revealed the existence of two chlorine atoms in the molecular structure of the compound and the m/z 73 piece implied the existence of silicon atoms. It was found that the compound at the peak of 22.40 min had a MW of 284 and the spectrum analysis (Figure 11) implied the existence of two chlorine atoms in its molecular structure. It could be deduced that the compound was a metabolite of dichlorinated naphthol after silane derivatization. The compound at the peak of 26.44 min had a MW of 372, and the spectrum analysis (Figure 12) revealed there were two chlorine substitutes in the molecular structure and it could be a metabolite of hydroxyl-dichlorinated naphthalene after silane derivatization. Table 1 presents detailed analysis of the spectra at peaks 22.40 min and 26.44 min for the two metabolites.

**Table 1:** Analysis of spectra peaked at 22.40 min and 26.44 min for two metabolites of 1,4-DCN.

Peak Time (min)	Peak	Clusters	m/z	Molecular Formula
22.4				
	a	284		C_13_H_14_OSiCl_2_(M)
	b	269	284-15	C_12_H_11_OSiCl_2_(M-CH_3_)
	c	233	269-36	C_12_H_10_OSiCl (M-CH_3_-HCl)
	d	219	234-15	C_11_H_8_OSiCl(M-CH_3_-Cl- CH_3_)
	e	198	234-36	C_12_H_10_OSi(M-CH_3_-Cl-HCl)
	f	175	219-44	C_9_H_4_SiCl(M-(CH_3_)_2_-Cl- CH_3_CHO)
	g	73		-O-Si-(CH_3_)_3_
26.44				
	a	372		C_16_H_22_O_2_Si_2_Cl_2_(M)
	b	357	372-15	C_15_H_19_O_2_Si_2_Cl_2_(M-CH_3_)
	c	284	372-16-72	C_13_H_13_OSiCl_2_ (M-O-Si-(CH_3_)_3_)

It is noted that during the degradation experiments by *Pseudomonas* sp. HY, the formation and degradation of some metabolites may lead to the occurrence and disappearance of a yellow color. According to Kim and Picardal [17], the opening of aromatic rings may lead to the formation of yellow color metabolites while some polychlorinated benzenes (PCBs) were investigated. However, to date there were no reports on the formation of yellow color metabolites during the metabolism of PCNs. In this study, the UV absorbance of the yellow color compounds was at 379 nm. Further research is needed to isolate and identify these compounds.

### 3.3. Metabolic Pathway of 1,4-DCN

**Figure 13:**
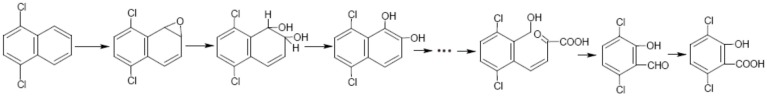
Metabolic pathway of 1,4-DCN.

The research suggests a metabolic pathway of 1,4-DCN based on available literatures on similar compounds and the analyses of chloride ions, metabolites and silane-derivatized metabolites of 1,4-DCN: (1) Monooxygenase reacts with the No. 5,6 or 7,8 carbon atoms of 1,4-DCN and produces 1,4-dichloro-7,8-epoxy-naphthalene or 1,4-dichloro-5,6-epoxy-naphthalene; (2) The successive hydroxylation reaction leads to the formation of dihydroxyl-dihydro-1,4-dichloro naphthalene; (3) The compound is then dehydrogenized, which produces dihydroxyl-1,4-dichloro naphthalene: (4) The aromatic rings open and form 3,6-dichlorinated salicylic acid or 2,5-dichlorinated salicylic acid. In the meanwhile, the dihydroxyl-dihydro-chlorinated naphthalene may be hydrolyzed, which produces naphtol compounds, in an acidic environment. The metabolic pathway, as shown in Figure 13, resembles that of naphthalene and that of PCBs, which have similar structures compared to 1,4-DCN [18,19,20].

## 4. Conclusions

The following conclusions were obtained from this study: (1) The 1,4-DCN can be biodegraded and the experiments using a bacterial strain *Pseudomonas* sp. HY revealed 98% removal of 1,4-DCN given sufficient time at certain initial concentrations; (2) The degradation of 1,4-DCN led to the formation of various metabolites including dihydroxy-dichloro-naphthalene, epoxy-dichlorinated naphthalene, dichlorinated naphthol, and dichlorinated salicylic acid; (3) The degradation may not remove chloride ions from the 1,4-DCN and its metabolites may not benefit the bacterial growth. The research suggests a metabolic pathway of 1,4-DCN, which is critical for the treatment of this compound through biological processes.
